# Global oral health status of athletes with intellectual disabilities

**DOI:** 10.1007/s00784-017-2258-0

**Published:** 2017-11-09

**Authors:** Luc Marks, Allen Wong, Steven Perlman, Amy Shellard, Carla Fernandez

**Affiliations:** 1 Dental School, Centre of Special Care in Dentistry, PaeCoMeDiS, Ghent University Hospital, Pintelaan 185, 9000 Ghent, Belgium; 2 Department of Dental Practice, University of the Pacific Arthur A. Dugoni School of Dentistry, San Francisco, CA USA; 3 Department of Pediatric Dentistry, Boston University, Boston, MA USA; 4 Special Olympics International, Washington, DC USA

**Keywords:** World health, Intellectual development disorder, Dental health survey, Treatment needs, Oral disease

## Abstract

**Background:**

The aim of this study is to identify the oral health status and treatment needs of Special Olympics athletes with intellectual disabilities from 181 countries by the assessment of oral health parameters and differences between world regions.

**Material and methods:**

Data were collected through interview and oral examinations within the Healthy Athletes Screening. These data were analysed with descriptive statistics of oral health parameters of athletes from Africa, Asia Pacific, East Asia, Europe/Eurasia, Latin America, Middle East North Africa (MENA) and North America. Mean differences of untreated visible dental caries, gingival signs and missing teeth were tested between regions by one-way ANOVA test and between age groups (8–11, 12–18, 19–39 and 40+) by chi-square tests for multiple comparisons with Hochberg-adjusted *p* value. The level of significance for all tests was set at a *p* value < 0.05.

**Results:**

A total of 149,272 athletes with intellectual disabilities were screened. More than 80% of the athletes reported that they cleaned their mouths at least once a day. Athletes in Europe/Eurasia, Latin America, and MENA presented higher rates of signs of gingival disease than other regions. The prevalence of untreated dental caries was significantly higher in Latin America and the group of 8–11-year-olds from Latin America, Europe/Eurasia and Asia Pacific.

**Conclusions:**

The data provided by this study demonstrate that continuous efforts for preventive and restorative oral health care are needed for the oral health of these athletes with ID especially in Latin America, MENA and Europe/Eurasia regions.

## Introduction

Oral health needs of an individual have an impact on the general health and quality of life. Dental caries and periodontal disease are the two most prevalent oral diseases and may compromise eating, speech and self-esteem [1, 2]. In addition, evidence has demonstrated that poor oral health is associated with malnutrition, weight loss, systemic diseases and focal infections, which may increase morbidity and mortality [3]. Regardless of the efforts to promote oral health as an integral part of total health, the global burden of oral disease has increased in the past 20 years, mainly as a consequence of population growth and people living longer [4]. Moreover, poverty has an important influence in the levels of oral disease and access to care as seen in developing nations with high rates of poverty. Therefore, oral health problems remain a public health challenge [4].

In relation to oral health issues, individuals with intellectual disabilities are among the most vulnerable populations [5, 6]. Anders and Davis [5] conducted a systematic review to analyse the differences in the oral health status between patients with intellectual disabilities and the neurotypical population. This review, published in 2010, identified 86 studies, of which only 27 met criteria. For inclusion, the studies involved adults with intellectual disability, applied at least one quantitative measure of oral health status and involved a control or comparison group without intellectual disability (ID) [5]. The review found enough evidence to suggest the finding that people with intellectual disabilities have higher plaque levels, poorer oral health and greater prevalence of periodontal disease but lower or similar prevalence of dental caries than the general population [5, 7–9]. According to Reid et al. [10], low prevalence of dental caries could be attributed to premature extraction of decayed teeth as a treatment choice as opposed to fillings, crowns or bridges.

Periodontal status of adults with intellectual disabilities is related to poor oral hygiene. Plaque index has been correlated with periodontal disease, and the indicators (probing depth and clinical attachment loss) were found to be higher in individuals with severe intellectual disabilities [11]. Therefore, it was concluded that periodontal status of the population with ID is related to poor oral hygiene and the potential need for periodontal treatment is greater for those with severe ID [5, 7, 11]. This may be attributed to a population that may not be able to comprehend the concept of personal oral hygiene techniques, and others may lack the dexterity to do it effectively. Further, the incidence of gingivitis in this population is 1.2 to 1.9 times the estimate for the general population [1, 12–14].

Clearly, oral health problems are far from being resolved, and even though people with intellectual disabilities are considered to be at a major risk, little has been done to eliminate the burden of oral disease in this population [4]. Large-scale international data on the oral health status of people with intellectual disabilities are scarce. These data could be important for the evaluation of existing policies, comparison of outcomes between countries and stimulation of international interventions and joint actions for health promotion and disease prevention.

The aim of the present study is to identify the oral health status and treatment needs of athletes with intellectual disabilities from 181 countries. For this purpose, several oral health parameters were assessed and differences between world regions regarding the prevalence of signs of gingival disease, untreated decay and missing teeth were reported as well as variations of those values among age groups.

## Methods

Oral health data were collected through interviews and oral examination of athletes participating in Special Olympics events from January 2007 to September 2015. The study participants attended the “Special Olympics Special Smiles” sites where they could have their teeth examined. Informed consent was obtained before every event in full accordance of the World Medical Association Declaration of Helsinki and in full accordance of the decision of the Joint Ethical Committee of the Ghent University 2013/816.

Data collection procedures included registration of the athletes’ demographic data (age, gender, country and date of birth), oral health screening and oral hygiene education. The oral screening consisted of the following parameters: edentulism, untreated dental caries, filled or missing teeth, sealants, tooth injury, fluorosis and signs of gingival disease [15].

In 1998, the Division of Oral Health, the National Center for Chronic Disease Prevention and Health Promotion and the Center for Disease Control and Prevention (CDC) developed the training manual for Standardized Oral Health Screening [15] to meet the needs of the Special Olympics Special Smiles program. The oral health screening was performed by dentists and dental hygienists, who were recruited from university dental schools and dental professional organizations. All examiners underwent training in the Special Smiles Standardized Oral Health data collection protocol [15].

The first question of the screening directed to the athlete was, “How often do you clean your mouth?” rather than, “How often do you brush your teeth?”; this was to record frequency of oral hygiene effort regardless of the specific devices used. In some parts of the world, other methods or devices other than toothbrushes are used to clean their mouths.

The second question was, “Do you have any pain inside your mouth now?” If the athlete answers, “yes”, they are asked to point where it hurts, teeth or any area of the oral cavity.

At this point, the athlete was asked “Can I look at your teeth today?” If he or she refused, data collection was terminated. If edentulous, the exam was completed; if not, the examination continued.

Untreated visible dental caries was recorded in both primary and permanent dentition (except third molars) when at least one area of cavitation that would accommodate a 0.5-mm-diameter (or larger) bur was detected. Any dental restorative work done exclusively as a response to dental caries was coded as “filled tooth”, and “missing tooth” was recorded if a tooth was not present at the time of the exam (with exception of premolars and third molars). Unerupted teeth were not counted as missing teeth.

For recording any evidence of dental trauma, only maxillary and mandibular central and lateral incisors in the permanent dentition were considered. This question was related to a tooth that was either absent, fractured or discoloured suggesting loss of vitality. Subsequently, the presence of sealants was recorded when material, placed as a preventive measure, covered the pits and fissures of the occlusal surfaces of only the first and/or second permanent molars.

The presence of fluorosis was noted when small, diffuse, opaque, paper-white areas and/or brown stains and pitting scattered were present over at least 25% of the buccal surface of maxillary front teeth (canine to canine). Signs of gingival disease were recorded if free or attached gingival margins or papillae showed significant deviations from normal colour, contour or texture on three or more permanent teeth in the mandibular buccal region from cuspid to cuspid.

Finally, treatment urgency was assessed based upon clinical findings. If there was no complaint of pain, no untreated dental caries or dental injuries and no signs of gingival disease, the examination was recorded for maintenance follow-up. In case of absence of pain, presence of dental caries but not involving the pulp, defective fillings and gingival problems without abscess formation, the athlete was referred for non-urgent treatment. In case of pain present inside the mouth, teeth with possible pulpal involvement, broken or missing fillings with caries or periodontal abscess formation, the athlete was referred for urgent treatment. After the examination, each athlete received individual oral health instruction adapted to his/her level of comprehension and manual dexterity.

**Fig. 1:**
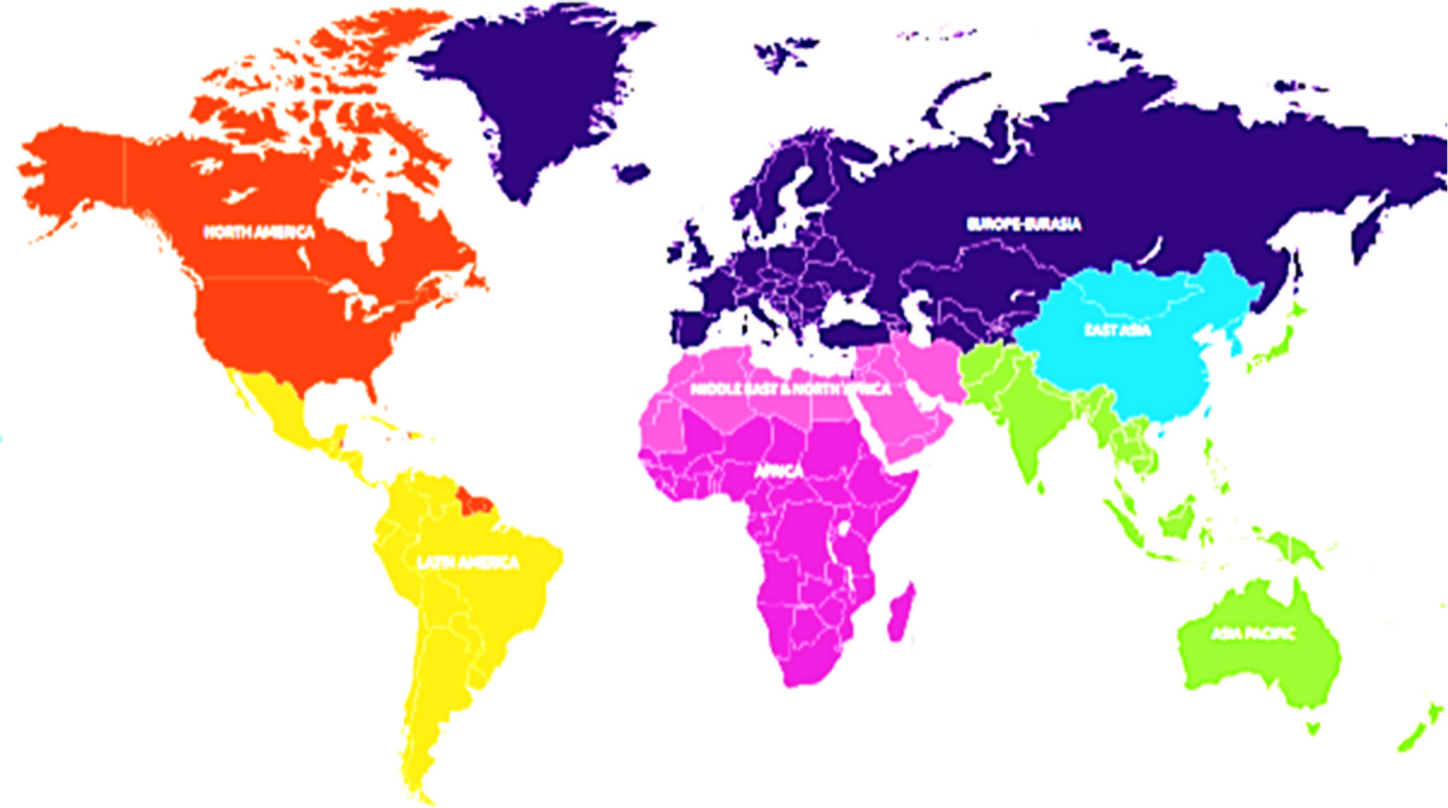
World regions according to classification of Special Olympics. (Extracted from Special Olympics Annual Report 2014) [16]. Africa (light purple), Asia Pacific (green), East Asia (light blue), Europe/Eurasia (dark purple), Latin America (orange), Middle East North Africa (pink) and North America (red)

All data collected manually were entered into the Healthy Athletes electronic data system and then downloaded into an Excel worksheet and transferred to an SPSS data file. Data analysis consisted of descriptive statistics of oral health parameters from countries grouped in regions, namely Africa, Asia Pacific, East Asia, Europe/Eurasia, Latin America, Middle East North Africa (MENA) and North America (Fig. 1). Differences in mean untreated dental caries, gingival signs and missing teeth were tested between regions by one-way ANOVA test. Finally, the global data were divided into four age groups (8–11, 12–18, 19–39 and 40+ years) and chi-square tests for multiple comparisons with Hochberg-adjusted *p* value were performed to assess differences of untreated dental caries, gingival signs and missing teeth between the regions per age group. For these analyses, the MENA region was excluded because the sample was quite small and its division per age groups made the comparisons impossible. The level of significance for all tests was set at a *p* value < 0.05.

## Results

A total of 149,272 athletes with intellectual disabilities were screened from Africa (29 countries), Asia Pacific (25 countries), East Asia (5 countries), Europe/Eurasia (61 countries), Latin America (20 countries), MENA (22 countries) and North America (19 countries). Data concerning the number of potential participants providing consent, the number of edentulous participants and the number of those who refused clinical examination per event and country are not available. Age and gender distribution is shown in Tables 1 and 2.

**Table 1:** Age distribution

	Number	Mean	Std. dev.	Min.	Max.	8–11	12–18	19–39	40+
Africa	11,323	19.3	12.74	8	88	18.7%	48%	26.6%	6.7%
Asia Pacific	12,945	23.1	13.91	8	85	11.6%	36.3%	39%	13.1%
East Asia	10,368	26.7	15.68	8	75	8.6%	30.6%	35.8%	25.1%
Europe/Eurasia	23,576	28.3	12.62	8	79	3.1%	26%	44.6%	26.3%
Latin America	16,331	25,3	11.46	8	86	10.9%	27.7%	45.2%	16.2%
MENA	850	19.5	5.86	8	53	5.9%	53.6%	39.3%	1.2%
North America	77,535	27.1	11.73	8	90	5.1%	20.5%	60.6%	13.8%

*MENA* Middle East North Africa

**Table 2:** Gender distribution

	Female	Male	Unknown			
	*n*	%	*n*	%	*n*	%
Africa	4220	37.3	7060	62.4	43	0.1
Asia Pacific	4272	33	8613	66.5	60	0.5
East Asia	3589	34.6	6719	64.8	60	0.6
Europe/Eurasia	8553	36.3	14,018	63.2	105	0.5
Latin America	6289	38.5	9994	61.2	48	0.3
MENA	303	35.7	497	58.5	50	5.9
North America	31,008	40	46,152	59.5	375	0.5

*MENA* Middle East North Africa

Demographic characteristics, reported oral hygiene habits and clinical parameters are presented in Table 3. Athletes from MENA and Latin America presented a higher rate of untreated dental caries and oral pain. Signs of gingival inflammation and referrals for urgent treatment were more prevalent in MENA, Europe and Latin America.

**Table 3:** Demographic characteristics, reported oral hygiene habits and clinical parameters

	Global	Africa	Asia Pacific	East Asia	Europe /Eurasia	Latin America	MENA	North America
	*n* = 149,272	*n* = 11,544	*n* = 13,814	*n* = 9207	*n* = 23,027	*n* = 17,821	*n* = 879	*n* = 72,849
	%	%	%	%	%	%	%	%
Mouth hygiene habits								
1. Less than once a week	1.5	3.7	0.9	0.3	2.7	1.1	4.8	1.1
2. Two to six times a week	9.8	14.2	7.8	3.8	10.0	13.2	16.1	9.4
3. Once a day	1.9	4.4	1.8	0.4	2.6	1.7	3.5	1.7
4. Twice or more a day	86.8	77.8	89.6	95.5	84.7	84.0	75.6	87.8
Mouth pain	14.4	20.4	17.4	8.4	11.5	25.9	21.3	11.8
Edentulous	1.4	0.6	0.9	0.2	1.0	1.1	0.8	2.0
Untreated tooth decay	36.6	37.7	45.5	37.5	45.9	64.0	61.7	24.7
Filling	49.8	8.6	24.9	30.2	58.2	34.5	35.2	64.7
Missing teeth	28.2	16.6	20.4	15.4	40.9	27.5	34.4	29.2
Tooth sealants	14.2	1.7	5.6	8.5	14.1	8.3	2.9	20.2
Dental injury	7.9	4.3	6.8	4.6	13.0	6.5	19.4	7.6
Fluorosis	7.5	6.6	5.8	2.6	3.6	9.8	4.5	9.4
Gingival signs	46.4	34.3	42.7	37.5	51.6	50.4	67.0	47.3
Urgent dental referral	13.9	19.9	17.3	4.9	20.4	22.8	25.4	9.3

More than 80% of the athletes reported that they cleaned their mouths at least once a day, with the exception of Africa and MENA. Dental injury was more prevalent in athletes from MENA and in second place among European athletes who also presented with a high prevalence of missing teeth. North American athletes presented with higher rates of sealants and fillings.

The differences in mean untreated dental caries and signs of gingival disease between all the regions were statistically significant with one-way ANOVA test (*F* = 14.175, *p* ≤ 0.001 for untreated dental caries and *F* = 5.752, *p* = 0.001 for gingival signs), while differences of mean missing teeth were not significant (*F* = 2.564 *p* = 0.05).

From the multiple comparisons of mean untreated dental caries, missing teeth and gingival signs of disease between the world regions per age group, the significant results are shown in Table 4.

**Table 4:**
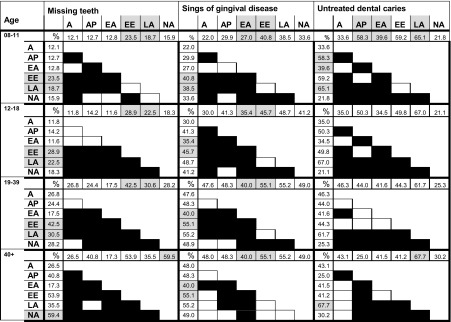
Multiple comparisons between regions per age group

Summary of multiple comparisons of pairwise chi-squared test statistics for differences in the prevalence of missing teeth, signs of gingival disease and untreated decay, among the different world regions per age group. The squares represent the Hochberg-adjusted *p* values of the difference between the compared values. Hochberg-adjusted *p* values < 0.05—black squares. MENA region was excluded from these analyses due to small sample size

*A* Africa, *AP* Asia Pacific, *EA* East Asia, *EE* Europe/Eurasia, *LA* Latin America, *NA* North America

The comparison of the different age groups (8–11, 12–18, 19–39 and 40+) shows that Europe/Eurasia and Latin America regions presented significantly more signs of gingival disease than other regions at all age groups. Further, the prevalence of this parameter was higher than 40% from the age of 13, in all regions. East Asia, on the other hand, presented significantly lower rates of gingival disease than Europe/Eurasia, Latin America and North America regions.

The prevalence of untreated dental caries was significantly higher in Latin America and significantly lower in North America. The group of 8–11 years showed higher prevalence of untreated dental caries especially in Latin America, Europe/Eurasia and Asia Pacific.

## Discussion

The analyses of the data revealed great variability in the distribution of oral disease between different regions of the world.

With the exception of Africa and MENA, more than 80% of the athletes reported that they cleaned their mouths at least once a day. This can be interpreted as proof of a certain level of knowledge regarding oral hygiene (practices). Although the effectiveness of oral cleaning was not a measurable requirement, the athletes’ cognitive and motor skills might compromise their ability to perform adequate personal oral hygiene. It was therefore recommended the supervision or assistance of a caregiver [1].

The prevalence of signs of gingival disease was considerable despite the high frequency of mouth cleaning reported. Existing literature reports show that gingivitis affects 28 to 75% of the general population worldwide [1, 3]; however, in this study its assessment was based only on the mandibular anterior region. The presence of gingival disease may be explained by inadequate brushing techniques, by motor and coordination impairments or by the specific presence of subgingival bacterial species and impaired immunological responses as in the case of individuals with Down syndrome [7, 17, 18].

The World Health Organization (WHO) has studied the burden of periodontal disease, among the general population [19]. The Community Periodontal Index was used for data collection; this index gives a score to qualify the periodontal conditions as healthy periodontal conditions, gingival bleeding, gingival bleeding and calculus, shallow periodontal pockets (4–5 mm) or deep periodontal pockets (‡ 6 mm). According to these data, the South-East Asia region was the region with higher prevalence of all the non-healthy periodontal conditions following America and Europe regions. Those results are comparable with our findings of signs of gingival inflammation in athletes with ID. Among the different age groups, the prevalence of gingival disease was higher than 40% from the age of 13 in all regions and Europe/Eurasia and Latin America were the most affected regions including all of the age groups.

Europe/Eurasia was also the region with the greatest prevalence of missing teeth in all age groups and only slightly surpassed by North America in the ages of 40+ years (53.4%EE; 59.5% NA) In the group of 12–18-year-olds, 29% of the athletes had already experienced tooth loss. Extraction seems to be frequently the treatment of choice for children and adults. In summary, prevention should be a priority for people with intellectual and physical disabilities to diminish and lower the prevalence of oral health problems.

Dental caries has been reported to affect 60–90% of school-aged children and the majority of adults in industrialized countries. In Asian and Latin American countries, it is the most prevalent oral disease while in African countries its prevalence has been reported to be lower [19]. The epidemiology of caries among the general population in different world regions was reported in a study based in WHO data from 12-year-old children. According to the results, the American region (North America and Latin America) and the Europe region presented a risk of 1.14 and 1.10 times higher than the average in the world. The African region was with a 19% lower risk compared to the average of all countries surveyed. In a big picture, those results agree with our findings [20]. The prevalence of untreated dental caries in athletes with ID among the different age groups (excluding MENA as mentioned in the “Methods” section) showed that, as previously discussed, Latin America and Europe/Eurasia have the highest prevalence. In Latin America, athletes aged 12–18 and those over 40 years presented the highest prevalence (67%), revealing that in this region dental caries is an important and universal unresolved health problem. The mean age of the participant athletes in all the regions ranged between 19 and 27 years. For this reason, the prevalence of caries and gingival disease becomes a concern, given the long-term negative impact of oral diseases on speech and nutrition, and general health. It must be pointed out that for the screening, the protocol focuses on the presence of untreated dental caries, but the number of teeth or severity of the condition is not reported. Secondly, it is a visual assessment that considers cavitation of at least 0.5 mm diameter; therefore, the actual burden of untreated dental caries among the athletes is expected to be higher [15, 21]. The use of other diagnostic tools such as radiographs and probes would increase the amount of the data collected.

The category of treatment urgency is a reflection of the severity of the athlete’s general oral condition. Regions with higher rates of referrals for urgent treatment were Europe/Eurasia 20.4%, Latin America 22.8% and MENA 25.4%. Referrals were made if the athletes presented with teeth with possible pulpal involvement, broken or missing fillings with caries, periodontal abscess formation or oral pain at the time of the screening. The prevalence oral pain (in one out of four athletes from Latin America) is a worrying statistic and would certainly affect the athletes’ ability to perform at his/her highest level.

Evidence has shown that water fluoridation reduces the prevalence of dental caries by 15% [22], and according to the WHO in many areas of the world, there are groundwaters with high fluoride concentrations, specifically in large parts of Africa, China, Eastern Mediterranean countries, Southern Asia and North America [23, 24]. Other countries employ community water or salt fluoridation [25–27]. As North America and Africa were the regions with the lowest scores of oral disease (gingival disease and untreated caries), further studies could elucidate where these results could be related to fluoride.

Diet and the consumption of food may be related as determining factor to the lower incidence of oral disease in athletes from developing countries [28, 29].

Further studies should help to elucidate other factors, which would explain these results, including a country’s policy of public and private health insurance, workforce of dental professionals and its infrastructure and methods of oral health surveillance.

Even though the protocol used has been widely accepted [21, 30–33], the study has some definite limitations that need to be considered when interpreting the results. First, given the population selected for this study, the results cannot be extrapolated to the entire population of individuals with ID. Athletes participating in SO belong to a highly supported but not necessarily any less dependent subgroup of the population with ID [21]. Additionally, the sample sizes obtained were convenience samples with a great variability of participants per country.

Athletes competing in Special Olympics would have performed at a level requiring some coordination and ability to follow some rules in order to qualify for regional events. The level of physical health in those screened at Special Olympic events is probably on the higher spectrum of developmental disability. Therefore, there is chance of selection bias.

Some questions, such as oral cleaning frequency and oral pain were answered by athlete self-reporting. Depending on the level of comprehension, the athletes may have given the answers that seemed appropriate to them, rather than what actually happens. Thereby, the frequency of mouth cleaning may be overestimated and/or the efficacy of the mouth cleaning may be inconsistent.

Following the Training Manual for standardized Oral Health Screenings [15], screeners are educated, trained and tested. However, no statistical analyses were performed and, therefore, they are trained examiners but not calibrated examiners. Therefore, and despite the screeners’ training, the fidelity with the criteria and adherence to the protocol may vary over time, as well as from region to region.

Global health is not only an aim but also a road that leads to fighting disparities and reducing vulnerability. Despite disparities, there has been an increase in globalization of research, innovations and expenditures. However, continuous efforts will be required by politicians, health care professionals, parents and carers to address oral health inequalities. For this purpose, the burden of oral diseases in each community must be individually assessed.

## Conclusion

The oral health status of athletes with intellectual disabilities from Africa, Asia Pacific, East Asia, Europe/Eurasia, Latin America, Middle East North Africa (MENA) and North America are different as a reflection of the different risk profiles of their countries and the influence of their health systems and preventive programs. The findings of this study were consistent with the trends of oral disease among the general population between different world regions. However, the consistency of the results demonstrates that within the limitations of this study, it can be concluded that there are high unmet-preventive and restorative oral health needs in athletes with intellectual disability. The fundamental challenge is to respond to the diversity of urgent oral health needs of this population by all major stakeholders. Specific challenges in this regard are especially important in all regions. Global reinforcement of oral health programs through the implementation of effective preventive measures for oral disease and oral health promotion has become an urgent need that must be addressed.

## References

[1] Horwitz SM, Kerker BD, Owens PL, Zigler E (2000). The health status and needs of individuals with mental retardation.

[2] Petersen PE (2003). The World Oral Health Report 2003: continuous improvement of oral health in the 21st century—the approach of the WHO Global Oral Health Programme. Community Dent Oral Epidemiol.

[3] Kandelman D, Petersen PE, Ueda H (2008). Oral health, general health, and quality of life in older people. Spec Care Dentist.

[4] Marcenes W, Kassebaum NJ, Bernabe E, Flaxman A, Naghavi M, Lopez A (2013). Global burden of oral conditions in 1990-2010: a systematic analysis. J Dent Res.

[5] Anders PL, Davis EL (2010). Oral health of patients with intellectual disabilities: a systematic review. Spec Care Dentist..

[6] Scott A, March L, Stokes ML (1998). A survey of oral health in a population of adults with developmental disabilities: comparison with a national oral health survey of the general population. Aust Dent J.

[7] Owens PL, Kerker BD, Zigler E, Horwitz SM (2006). Vision and oral health needs of individuals with intellectual disability. Ment Retard Dev Disabil Res Rev.

[8] Hennequin M, Moysan V, Jourdan D, Dorin M, Nicolas E (2008). Inequalities in oral health for children with disabilities: a French national survey in special schools. PLoS One.

[9] Tesini DA, Fenton SJ (1994). Oral health needs of persons with physical or mental disabilities. Dent Clin N Am.

[10] Reid BC, Chenette R, Macek MD (2001). Prevalence and predictors of untreated caries and oral pain among Special Olympic athletes. Spec Care Dentist..

[11] Ozgul O, Dursun E, Ozgul B, Kartal Y, Coskunses F, Kocigyt I (2014). The impact of handicap severity on oral and periodontal status of patients with mental retardation. J Contemp Dent Pr.

[12] Hennequin M, Faulks D, Roux D (2000). Accuracy of estimation of dental treatment need in special care patients. J Dent.

[13] Vignehsa H, Soh G, Lo GL, Chellappah NK (1991). Dental health of disabled children in Singapore. Aust Dent J.

[14] Cumella S, Ransford N, Lyons J, Burnham H (2000). Needs for oral care among people with intellectual disability not in contact with Community Dental Services. J Intellect Disabil Res.

[15] White JA, Beltran ED. (2004) Training manual for standardized oral health screening training manual for standardized oral health Screening Available from: http://media.specialolympics.org/resources/health/disciplines/specialsmiles/Special-Smiles-Training-Manual.pdf

[16] Special Olympics Annual Report 2014. Available from: http://media.specialolympics.org/resources/reports/annual-reports/2014_AnnualReport-full.pdf

[17] Sakellari D, Arapostathis KN, Konstantinidis A (2005). Periodontal conditions and subgingival microflora in Down syndrome patients. A case-control study. J Clin Periodontol.

[18] Khocht A, Janal M, Turner B (2010). Periodontal health in Down syndrome: contributions of mental disability, personal and professional dental care. Spec Care Dentist..

[19] Petersen PE, Ogawa H (2012). The global burden of periodontal disease: towards integration with chronic disease prevention and control. Periodontol.

[20] Moreira R da S (2012) Epidemiology of dental caries in the world. Res Epidemiol Clin Pract 149–168. 10.5772/31951

[21] Feldman CA, Giniger M, Sanders M, Saporito R, Zohn HK, Perlman SP (1997). Special Olympics, special smiles: assessing the feasibility of epidemiologic data collection. J Am Dent Assoc.

[22] McDonagh MS, Whiting PF, Wilson PM, Sutton a J, Chestnutt I, Cooper J (2000). Systematic review of water fluoridation. BMJ.

[23] Fawell J, Bailey K, Chilton J, Dahi E, Fewtrell L, Magara Y. (2006) Fluoride in Drinking-water. www.who.int/water_sanitation.../fluoride_drinking_water_full.pdf

[24] Palmer CA, Gilbert JA (2012). Position of the academy of nutrition and dietetics: the impact of fluoride on health. J Acad Nutr Diet.

[25] Jones S, Burt BA, Petersen PE, Lennon MA (2005). The effective use of fluorides in public health. Bulletin of the World Health Organization. Bull World Health Organ.

[26] Kargul B, Caglar E, Tanboga I (2003). History of water fluoridation. J Clin Pediatr Dent.

[27] Pizzo G, Piscopo MR, Pizzo I, Giuliana G (2007). Community water fluoridation and caries prevention: a critical review. Clin Oral Investig.

[28] Popkin BM, Adair LS, Ng SW (2012). Global nutrition transition and the pandemic of obesity in developing countries. Nutr Rev.

[29] Thorpe S (2006). Oral health issues in the African region: current situation and future perspectives. J Dent Educ.

[30] Fernandez C, Kaschke I, Perlman S, Koehler B, Marks L (2015) A multicenter study on dental trauma in permanent incisors among Special Olympics athletes in Europe and Eurasia. Clin Oral Investig. 10.1007/s00784-015-1403-x 10.1007/s00784-015-1403-x 25600092

[31] Turner S, Sweeney M, Kennedy C, Macpherson L (2008). The oral health of people with intellectual disability participating in the UK Special Olympics. J Intellect Disabil Res.

[32] Trihandini I, Wiradidjaja Adiwoso A, E rri Astoeti T, Marks L (2013). Oral health condition and treatment needs among young athletes with intellectual disabilities in Indonesia. International J Paediatr Dent.

[33] Oredugba FA, Perlman SP (2010). Oral health condition and treatment needs of Special Olympics athletes in Nigeria. Spec Care Dentist..

